# Percutaneous endoscopic interlaminar discectomy via inner border of inferior pedicle approach for downmigrated disc herniation: a retrospective study

**DOI:** 10.1186/s13018-022-03245-8

**Published:** 2022-07-21

**Authors:** Huiyu Huang, Haigang Hu, Xu Lin, Chao Wu, Lun Tan

**Affiliations:** 1 Department of Spine Surgery and Traumatology Surgery, Zigong Fourth People’s Hospital, Zigong, 643000 Sichuan People’s Republic of China; 2 Department of Spine Surgery, Affiliated Hospital of Southwest Medical University, Luzhou, 646000 Sichuan People’s Republic of China

**Keywords:** Percutaneous endoscopic interlaminar discectomy, Migrated disc herniation, Minimally invasive spine surgery

## Abstract

**Objective:**

To evaluate the efficacy and feasibility of percutaneous endoscopic interlaminar discectomy (PEID) via the inner border of the inferior pedicle approach for downmigrated disc herniation.

**Methods:**

Seventeen patients who had downmigrated disc herniation were included in this study from May 2020 to February 2021. After PEID via the inner border of the inferior pedicle approach, a retrospective study was conducted on all patients. Radiologic findings were investigated, and based on the level of migration seen on preoperative magnetic resonance imaging (MRI), participants were divided into two types (high-grade and low-grade migrations). Preoperative, 1st post-operative day, 3rd post-operative month, and the final follow-up visual analogue scale (VAS) assessments for back and leg pain and preoperative, 3rd post-operative month, and the final follow-up Oswestry disability index (ODI) evaluations were performed. The clinical effects at the final follow-up were assessed by the modified MacNab criterion.

**Results:**

All patients successfully completed surgery. There were 10 males and 7 females in the group. These patients were 42 years old on average (range, 25–68 years). Four and 13 patients had downmigrated disc herniation with high-grade and low-grade, respectively, on MRI. The mean follow-up duration was 10.47 ± 1.84 months (range, 8–14 months). The mean VAS score for back and leg improved from 5.18 ± 0.81 preoperatively to 1.35 ± 0.49 at the final follow-up (*P* < 0.05) and 6.94 ± 0.66 preoperatively to 1.47 ± 0.51 at the final follow-up (*P* < 0.05), respectively. The mean ODI score improved from 48.00 ± 3.64 preoperatively to 18.71 ± 1.31 at the final follow-up (*P* < 0.05). According to the modified MacNab criterion, 15 patients (88.2%) obtained excellent, while the rest 2 patients (11.8%) reported good outcomes.

**Conclusion:**

PEID via the inner border of the inferior pedicle approach could be a good alternative option for the treatment of downmigrated disc herniation.

## Introduction

Percutaneous endoscopic lumbar discectomy (PELD) has become a prominent treatment procedure for lumbar disc herniation (LDH) due to advancements in minimally invasive surgical procedures [1]. Specifically, PELD has various advantages over traditional surgical discectomy, including a speedier recovery, a smaller skin incision with less scarring and muscle damage, a lower infection rate, a less painful procedure, and a shorter hospital stay [2, 3].

However, the treatment of downmigrated disc herniation remains clinically complicated due to anatomic obstacles and disc fragmentation [4]. This is especially true if the downmigrated disc herniation has moved far (i.e. medial to the pedicle, beneath the pars interarticularis). Even for experienced spinal surgeons, deciding on a surgical procedure for these patients is more difficult. Although the treatment of downmigrated disc herniation has demonstrated PELD feasibility in previous studies, the failure rate is very high (5–22%) [4]. However, Krzok et al. [5] showed a new percutaneous endoscopic technique that creates a tunnel through the pedicle to reach its medial wall, in which the downmigrated disc herniation can be removed. Similarly, for transforaminal endoscopic discectomy, Kim et al. [6] developed a suprapedicular circumferential opening approach that involves drilling the articular process, upper pedicle, and upper-posterior edge of the lower vertebra to widen the foramen and expose the ventral epidural region. They obtained good to exceptional clinical results for high-grade downmigrated disc herniation.

However, downmigrated disc herniation has been treated by modified PELD techniques, with good clinical results. These methods still have a few shortcomings and limitations. We describe the details of a unique inner border of the inferior pedicle approach performed with the fenestration laminectomy technique using trephine under C-arm and visualization control and demonstrate the clinical results and efficacy in limited indications of PEID.

## Materials and methods

### Inclusion criteria, exclusion criteria and patients

The inclusion criteria were as follows: (1) preoperative MRI and CT scan revealed a single-level (L4/5 or L5/S1) downmigrated herniation and accompanying nerve root compression in patients with back and leg discomfort; (2) straight leg raising test was positive that the angle less than 50°; (3) no previous history of lumbar surgery at the same level; and (4) failure of conservative therapies over 4–6 weeks.

The exclusion criteria were as follows: (1) central stenosis confirmed by MRI and CT; (2) clear instabilities or deformities; and (3) infections, tumours, and fractures are examples of diseases or injuries that impact the spine.

Based on the inclusion and exclusion criteria, 17 patients who had downmigrated disc herniation were included in this study from May 2020 to February 2021. There were 10 males and 7 females in the group. These patients were 42 years old on average (range, 25–68 years). Four and 13 patients had downmigrated disc herniation with high-grade and low-grade lesions, respectively, on MRI. The mean follow-up duration was 10.47 ± 1.84 months (range, 8–14 months). Written informed consent was obtained from all patients. The study was accepted by the Fourth People's Hospital of Zigong affiliated with Southwest Medical University. All patients were successfully operated on by a well-trained and experienced spine surgeon in our hospital. The clinical characteristics are summarized in Table 1.

**Table 1:** Characteristics and operation-related data of the included patients (*n* = 17)

Variables	Results
Gender (male/female) (*n*)	10:7
Age (median, range) (year)	42 (25,68)
Target level (L4/5:L5/S1) (*n*)	9:8
Radiologic classification (low grade/high grade) (*n*)	13:4
Operation time (mean ± SD) (min)	87.65 ± 16.69
Follow-up time (mean ± SD) (month)	10.47 ± 1.84

### Radiologic classification

**Fig. 1:**
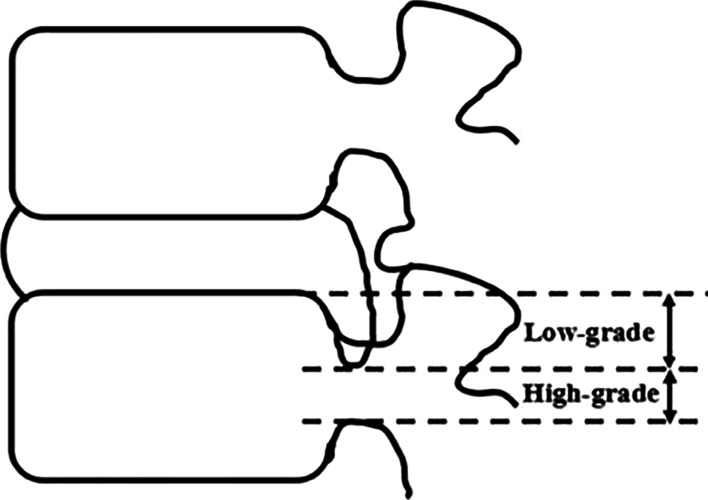
Schematic representation of the extent of the downmigrated herniation about the posterior height of the disc space [9]

Downmigrated herniation was located away from the extrusion site below the superior endplate level of the inferior vertebra, according to preoperative sagittal MRI [7]. If the extent of migration on T2-weighted sagittal MRI exceeded the height of the posterior marginal disc space, the herniation was classified as high-grade downmigrated disc herniation. Low-grade downmigrated disc herniation, on the contrary, was defined as migration whose extent was less than the height of the posterior marginal disc space [8] (Fig. 1).

### Surgical procedures

**Fig. 2:**
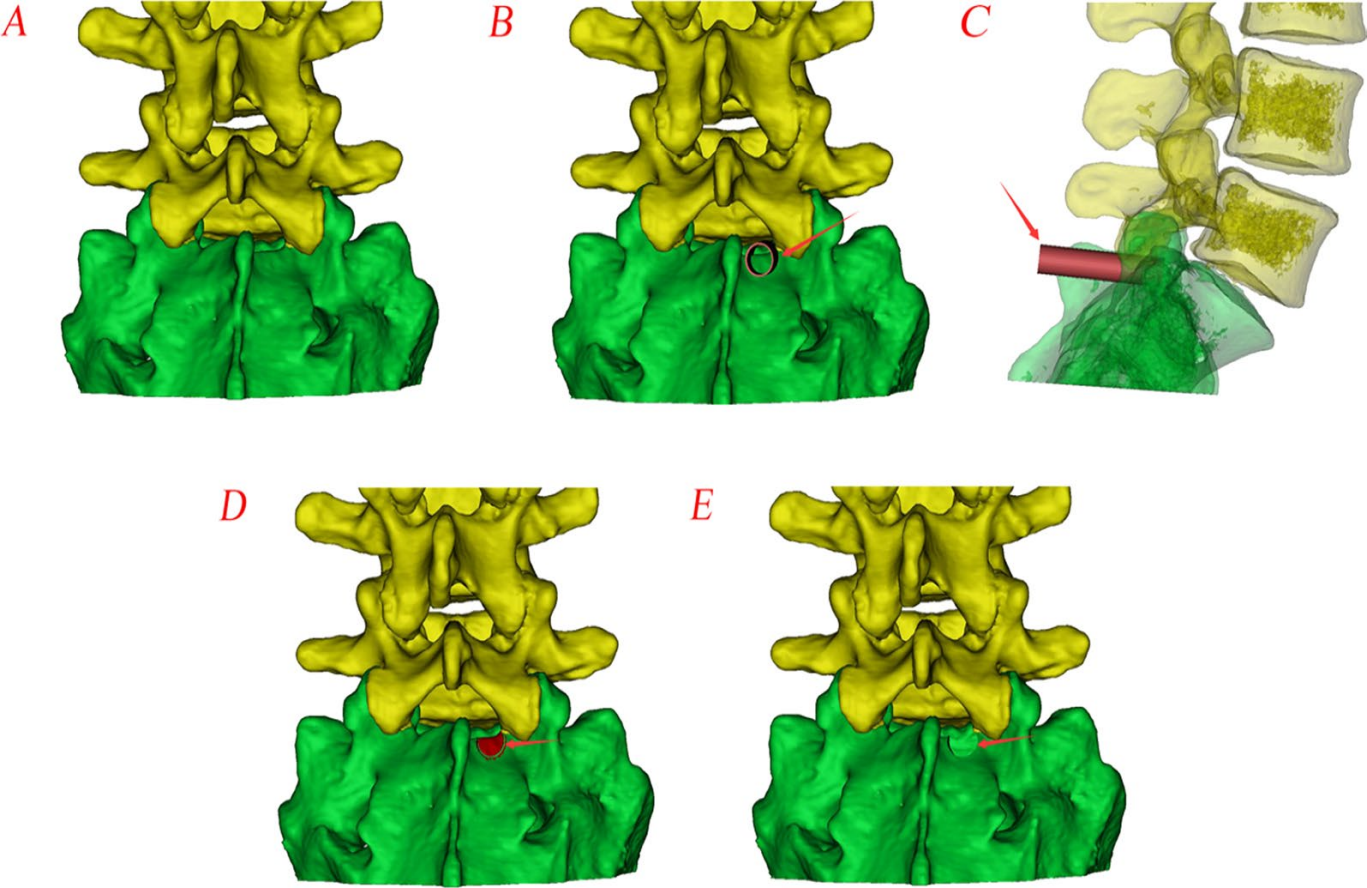
Diagrams of fenestration laminectomy. **A** Spine model; **B**, **C** location of the trephine under anterior–posterior fluoroscopy (arrow: trephine); **D** area (red part) cut off and removed together with the trephine; **E** after fenestration laminectomy

The fenestration laminectomy diagrams of PEID via the inner border of the inferior pedicle approach for downmigrated disc herniation are shown in Fig. 2.

#### Step 1. Anaesthesia and position

General anaesthesia was used for the PEID procedure. Patients were positioned in the prone decubitus posture on a radiolucent operating table, with a pillow placed between the lower abdomen and chest to allow the abdomen to freely suspend, thereby widening the interlaminar window.

#### Step 2. Approach

**Fig. 3:**
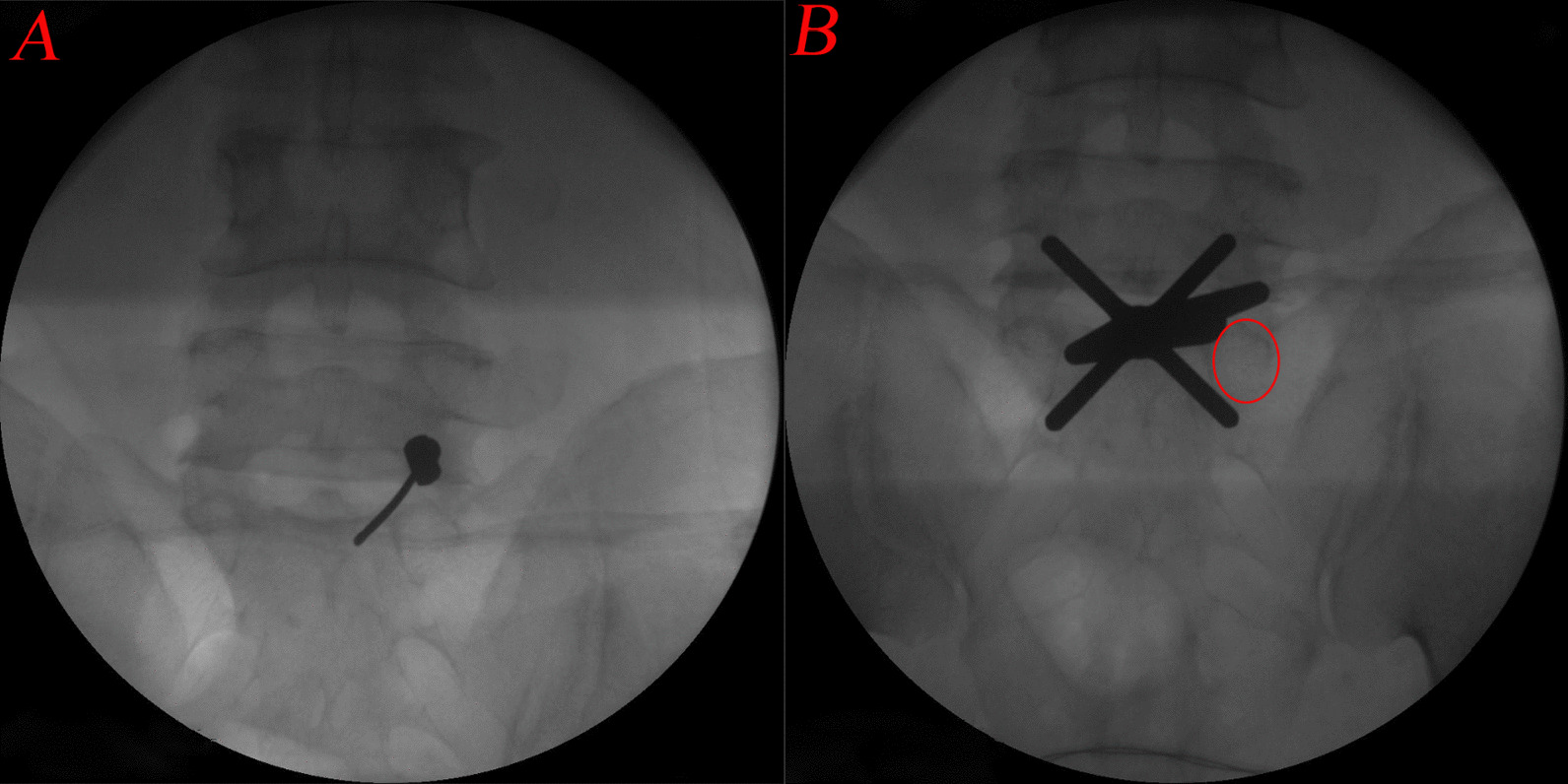
**A** Intraoperative fluoroscopy of the puncture needle;** B** Intraoperative fluoroscopy of the trephine (circle: the pedicle projection)

Anterior–posterior (AP) and lateral fluoroscopy were used to validate the skin entry point target level. The puncture needle was inserted from the marked skin entry point to the inner border of the inferior pedicle, which needed to be reconfirmed by AP fluoroscopy (Fig. 3A). A series of dilators were advanced to the surface of the inner border of the inferior pedicle after an 8-mm incision was made alongside the spinous process. Then, the operative sheath and trephine were introduced along the dilators. After introducing the endoscope, the soft tissue and paraspinal muscles were cleared to expose the inner border of the inferior pedicle. In addition, the operative sheath and trephine should be confirmed by AP fluoroscopy again to avoid damaging the medial wall of the pedicle (Fig. 3B). The procedure was performed under a continuous normal saline irrigation system.

#### Step 3 Fenestration laminectomy

**Fig. 4:**
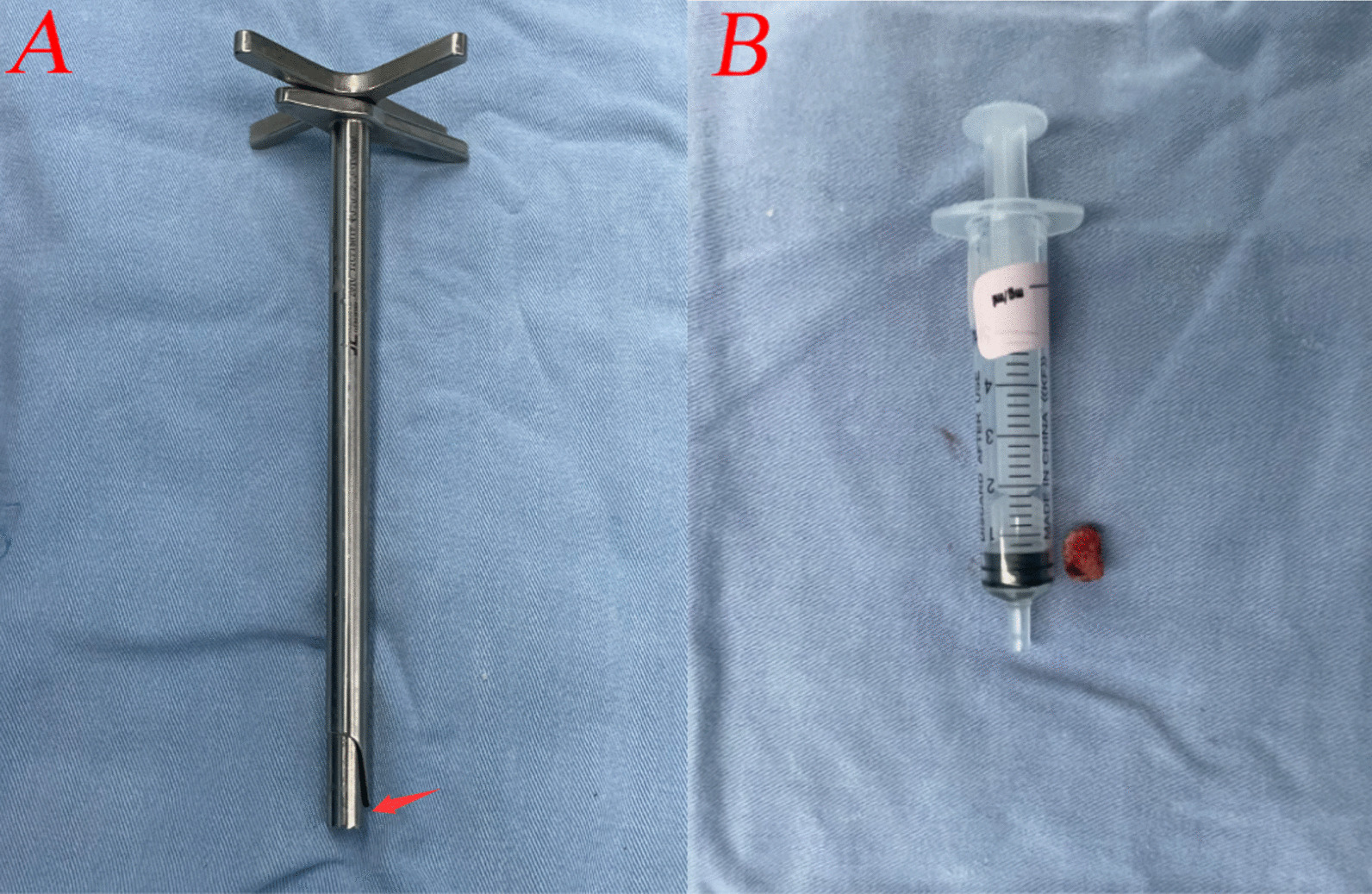
**A** The limited trephine depth; **B** the lamina bone was cut off by the trephine

**Fig. 5:**
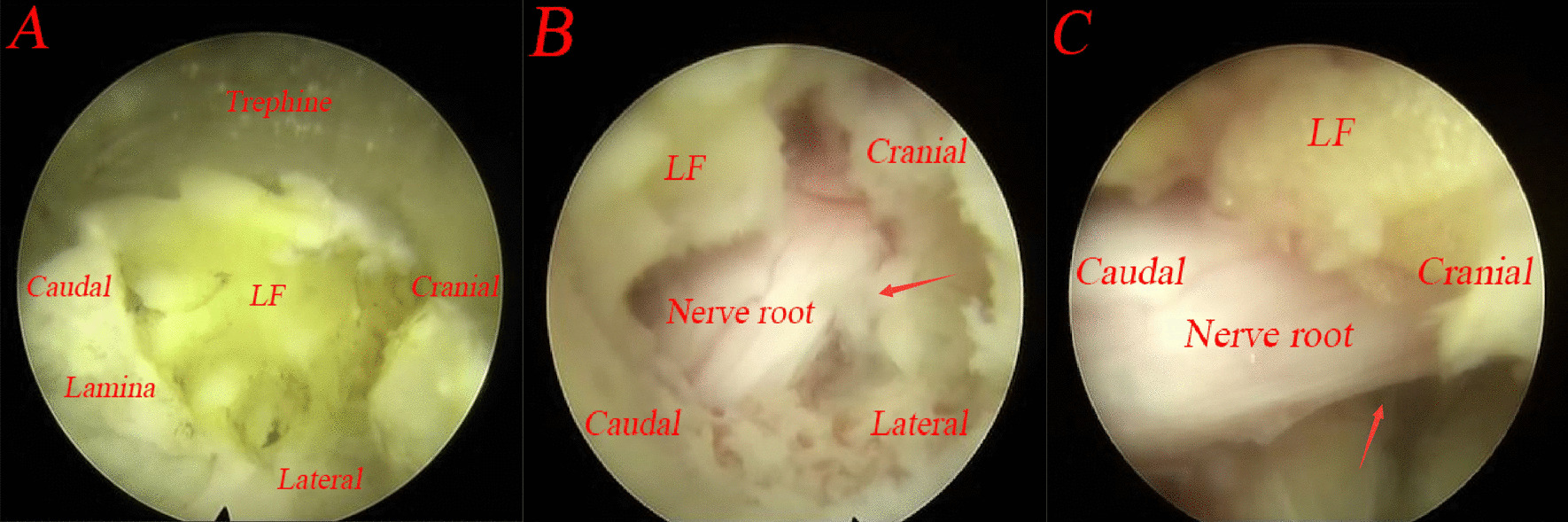
The operation is performed under endoscopy. **A** The trephine under endoscopy; **B** exposed nerve root (arrow: the tail of the downmigrated disc herniation); **C** complete decompression

The trephine was used to expand the interlaminar window during fenestration laminectomy. With cautious rotation under clear endoscopic visibility (Fig. 5A), the trephine depth should be limited (Fig. 4A). The lamina bone was cut off and removed together with the trephine (Fig. 4B), in some cases with a tiny part of the medial facet joint removed as well. If necessary, fenestration laminectomy can be repeated, and the laminar rongeur can be used to repair the lamina edges. To gain access to the epidural area after fenestration laminectomy, a tiny proportion of the ligamentum flavum was cut away with scissors. The nerve root and the tail of the downmigrated disc herniation were completely revealed on the endoscope (Fig. 5B).

#### Step 4. Decompression

After the structures have been confirmed under clear endoscopic visualization, the visible and downmigrated disc herniation should be first grabbed as much as possible and removed as a whole to reduce nerve root tension. It could decrease the chance of nerve root damage when the lateral border of the nerve root was pushed and thus protected by manoeuvring the operative sheath. Additionally, the ventral and axillary parts of the nerve root that the fragments could be easily removed. Then, debridement of the intervertebral space was performed. Finally, to control bleeding and ablate the disc, plasma radiofrequency was used. When the nerve root had been examined and released, the operation was finished (Fig. 5C). No drainage was needed.

### Data collection

To evaluate the safety of the surgery, the operation time and complications during and post-operation were recorded. Preoperative, 1st post-operative day, 3rd post-operative month, and final follow-up visual analogue scale (VAS) assessments for back and leg pain and preoperative, 3rd post-operative month, and final follow-up Oswestry disability index (ODI) evaluations were performed. The clinical effects at the final follow-up were assessed by the modified MacNab criterion. Assessments were completed by an independent observer.

### Statistical analysis

All statistical analyses were performed with SPSS v21.0 software (SPSS Inc., Chicago, IL, USA). Continuous variables were compared, such as VAS-Back, VAS-Leg, and ODI, by Bonferroni statistical analysis between different points in time. The mean and standard deviation are used to express the data. The test level was *α* = 0.05, and the statistical significance threshold was *p* < 0.05.

## Results

### Surgical information

All patients successfully completed surgery, and the pain in the back and leg was significantly relieved after the surgery, which demonstrated that the operation was technically feasible for all patients. The mean operative time was 87.65 ± 16.69 min (range, 60–115 min). There was no obvious measurable blood loss.

### Clinical outcomes

**Fig. 6:**
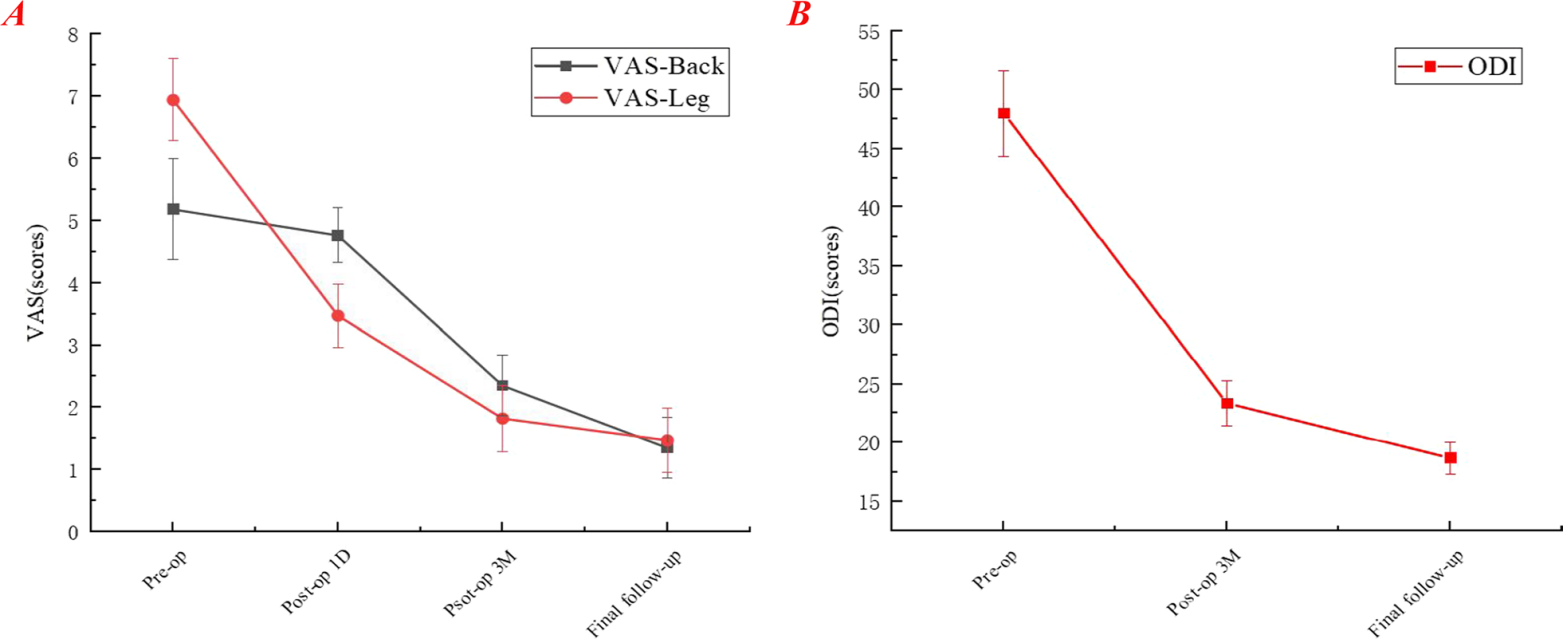
Clinical status and outcomes. **A** VAS for back and leg pain. **B** Oswestry disability index

Compared with the preoperative state, the VAS-Back, VAS-Leg, and ODI scores were significantly decreased at each post-operative time point (*P* < 0.05), except on the 1st post-operative day of VAS-Back (*P* > 0.05). The results of the VAS-Back, VAS-Leg, and ODI are shown in Table 2 and Fig. 6. According to the modified MacNab criteria, 15 patients (88.2%) obtained excellent outcomes, while the remaining 2 patients (11.8%) reported good outcomes (Table 3).

**Table 2:** Pain relief and functional improvement

Variables	Preoperative	1st Post-operative day	3rd Post-operative month	The final follow-up
VAS-Back	5.18 ± 0.81	4.76 ± 0.44	2.35 ± 0.49*	1.35 ± 0.49*
VAS-Leg	6.94 ± 0.66	3.47 ± 0.51*	1.82 ± 0.53*	1.47 ± 0.51*
ODI	48.00 ± 3.64	-	23.35 ± 1.93*	18.71 ± 1.31*

^*^P < 0.05 represents a statistical difference from preoperative data

**Table 3:** Modified MacNab criteria

	Excellent	Good	Fair	Poor
Patients (*n*)	15	2	0	0
Percentage (%)	88.2	11.8	0	0

### Radiological findings

**Fig. 7:**
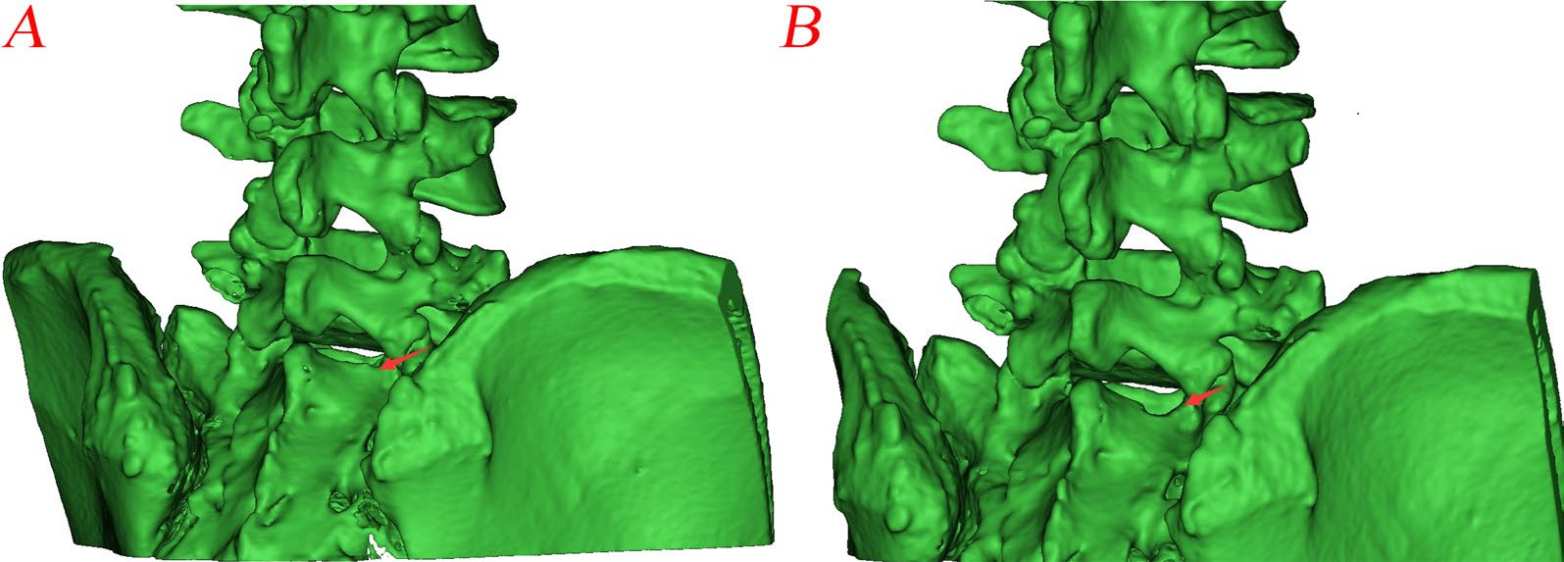
**A** Preoperative CT scan reconstruction;** B** Postoperative CT scan reconstruction

The bone resection of the laminar channel is similar to the preoperative design of fenestration laminectomy diagrams on post-operative reconstructed CT scans (Figs. 2E and 7).

**Fig. 8:**
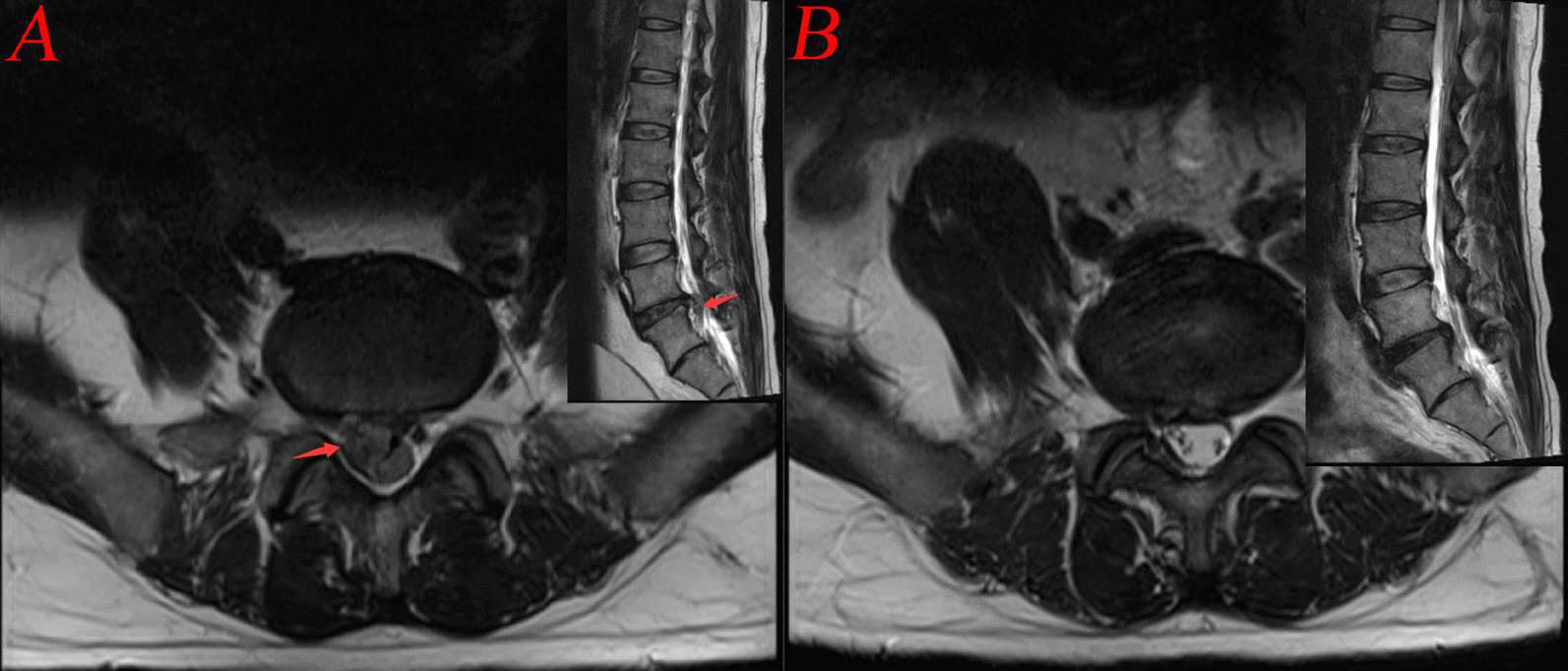
**A** Preoperative MRI (arrow: downmigrated disc herniation);** B** Postoperative MRI

MRI examinations in the third post-operative month revealed complete decompression of the downmigrated disc herniation, as well as the compressional dural sac and the shifting nerve root (Fig. 8).

### Complications

In our study, there were no occurrences of neurologic damage or cerebrospinal fluid leakage, although two patients experienced post-operative paresthesias that subsided in 4 to 6 weeks and were gone by the time of the final follow-up.

## Discussion

LDH is a prevalent cause of lower back and leg pain, with downmigrated disc herniation occurring in 35–72% of cases [10], which results in medical and financial consequences for families and society. The incidence of LDH is increasing as a result of our modern sedentary lifestyle. Conservative therapy is generally ineffective in these circumstances, thus necessitating surgical surgery. Currently, PELD, which includes PETD and PEID, has become the most popular approach for the treatment of LDH. PETD is a complicated operation with a steep learning curve. Multiple punctures and correct foraminoplasty are needed, especially when foraminal stenosis and a high iliac crest are present, which not only prolongs the surgical and fluoroscopy times but also increases the risk of nerve root damage [11]. A high iliac crest, a limited disc space, and overuse of radiography examinations of the lateral transforaminal approach might all be avoided by using PEID [12]. Moreover, it is more consistent with the surgeon's operating habits. However, it is also limited to patients with an interlaminar window narrow. Although advances in minimally invasive surgery have broadened the indications for PELD, the treatment of downmigrated disc herniation with either procedure is clinically challenging [13].

Although downmigrated disc herniation is traditionally considered difficult to treat with PELD, spinal surgeons have never ceased researching this technique because of its various benefits. During the operation, the disc herniation must be removed under clear visibility control to ensure complete decompression. This is important for surgical success, especially downmigrated disc herniation. Therefore, to create an optimum minimally invasive spinal surgical approach, it is necessary as close as possible to the downmigrated disc herniation or directly target the surgical objective. Thus, some surgeons use aiguille to create a bone tunnel in the pedicle or lamina to treat downmigrated disc herniation [5, 14]. However, even minor system movements are strongly prohibited when the endoscope is inside the bone tunnel, which may increase pedicle fracture risk. Additionally, a pedicle diameter of less than 8 mm is an absolute contraindication. Similar to the hole in the lamina [14, 15], due to the limitation of the bone tunnel, the scope of visual control cannot be enlarged with an inclined introduction and pivoting action of the working canal or the endoscope. A modified transforaminal PELD approach has been reported [6, 16, 17], in which the facet or pedicle is partially removed to widen the intervertebral foramen and reveal downmigrated disc herniation. However, more bony structures must be removed, especially for the treatment of highly downmigrated disc herniation (i.e. medial to the pedicle, beneath the pars interarticularis) that is hidden from the endoscopic view by anatomic barriers, and this approach may increase the risk of segmental instability in the future.

To acquire direct and adequate exposure of the nerve root and downmigrated disc herniation, a precise and universality hole on the inner border of the inferior pedicle was established by the trephine to assist PEID. After clinical application, this approach could be a safe and effective addition to PELD for downmigrated disc herniation. Additionally, as a fenestration laminectomy technology, the same is suitable for posterior epidural migration of intervertebral disc herniation [18]. This approach has several advantages: (1) Precision and universality lamina drilling of the hole eliminate the risk of segmental instability resulting from massive removal of articular processes as a rapid and appropriate method of fenestration laminectomy. It is suitable for most patients. (2) This surgical procedure could provide greater room for operation. Additionally, it is more consistent with the surgeon's operating habits. (3) Based on the larger operating space, the scope of the visual control can be easily enlarged with an inclined introduction and pivoting action of the working canal or the endoscope. (4) The most significant benefit is that this surgical method lowers epidural scarring, which is advantageous for performing revision procedures.

Based on existing clinical applications, we summarized several considerations for this procedure. (1) The operative sheath and trephine should be reconfirmed by anterior–posterior (AP) fluoroscopy to avoid damage to the medial wall of the pedicle. (2) The trephine depth should be limited, with cautious rotation under clear endoscopic visibility. (3) Bone bleeding during fenestration laminectomy can be considerable, but it can be controlled with the use of bipolar coagulation or by improving the pressure of the normal saline irrigation system.

However, in the current study, PEID via the inner border of the inferior pedicle approach also has some complications yet now, such as post-operative paraesthesia. Two patients experienced post-operative paresthesias that subsided in 4 to 6 weeks and were gone by the time of the final follow-up. According to our observations, post-operative paraesthesia may be caused by surgical injury of the nerve root. Additionally, post-operative dysesthesia may be exacerbated by repeated bipolar coagulation and extrusion of the working canal. According to the modified MacNab criterion, 15 patients (88.2%) obtained excellent outcomes, while the remaining 2 patients (11.8%) reported good outcomes. Compared with the preoperative state, the VAS-Back, VAS-Leg, and ODI scores were significantly decreased at each post-operative time point (*P* < 0.05), except on the 1st post-operative day of VAS-Back (*P* > 0.05). It has achieved a good clinical effect. Additionally, this is consistent with previous literature reports [19–21]. This approach was shown to be a safe and effective minimally invasive technique.

## Conclusion

Based on the terms of clinical outcomes, PEID via the inner border of the inferior pedicle approach demonstrated clinical results and efficacy in limited indications of PEID due to the high success rate and low complication rate. Finally, PEID via the inner border of the inferior pedicle approach could be a good alternative option for the treatment of downmigrated disc herniation.

### Limitation

In this study, there were several limitations. First, this was a retrospective study, and there was no control group, which made it difficult to determine whether this approach was superior. Moreover, the sample size was small, and the follow-up duration was short, which could have an impact on the results. Further studies with prospective, multicentre, large samples, control studies, and long-term follow-up will be conducted in the future. In addition, we did not conduct a post-operative flexion/extension correlation analysis during the follow-up.

## Data Availability

The data sets used and analysed during the current study are available from the corresponding author on reasonable request.

## Abbreviations

VAS: Visual analogue scale
ODI: Oswestry disability index
MRI: Magnetic resonance imaging
CT: Computed tomography
PELD: Percutaneous endoscopic lumbar discectomy
PEID: Percutaneous endoscopic interlaminar discectomy
LDH: Lumbar disc herniation
SD: Standard deviation
LF: Ligamentum flavum
AP: Anterior–posterior
